# The effect of user interface on treatment engagement in a self-guided digital problem-solving intervention: A randomized controlled trial

**DOI:** 10.1016/j.invent.2021.100448

**Published:** 2021-08-20

**Authors:** Amira Hentati, Erik Forsell, Brjánn Ljótsson, Viktor Kaldo, Nils Lindefors, Martin Kraepelien

**Affiliations:** a Center for Psychiatry Research, Department of Clinical Neuroscience, Karolinska Institutet, Stockholm Health Care Services, Region Stockholm, Stockholm, Sweden; b Division of Psychology, Department of Clinical Neuroscience, Karolinska Institutet, Stockholm, Sweden; c Department of Psychology, Faculty of Health and Life Sciences, Linnaeus University, Växjö, Sweden

**Keywords:** User interface, Treatment engagement, Self-guided, Digital intervention, Problem-solving, User experience

## Abstract

**Background:**

Resources are spent worldwide on the development of digital platforms and their user interfaces (UIs) for digital mental health services (DMHS). However, studies investigating the potential benefits of different UIs for DMHS are currently lacking. To fill this knowledge gap, the aim of this study was to investigate differences in treatment engagement between two different UIs for DMHS.

**Methods:**

A total of 397 participants from the Swedish general public were randomized (1:1) to use a self-guided digital problem-solving intervention during one week, either with an optimized UI (*N* = 198), based on user experience (UX) design principles and with automated features, or a basic UI (*N* = 199), analogous with a UI used in Swedish regular health care comprising elementary UI features and less automation. Primary outcome measures were self-rated usability, on the System Usability Scale, and treatment credibility, on the Credibility/Expectancy Questionnaire. Secondary outcome measures included behavioral engagement with the intervention.

**Findings:**

There were no significant differences between the UIs in ratings of usability or treatment credibility. However, participants who used the optimized UI were significantly more engaged with the intervention as measured by usage of the intervention at least once (odds ratio 2.54, 95% CI [1.67, 3.85]), total number of generated solutions (mean difference 1.41, 95% CI [0.72, 2.11]), and mean number of generated solutions per initiated problem-solving attempt (mean difference 1.45, 95% CI [1.06, 1.85]). Other findings included participants using the optimized UI rating the intervention as easier to understand, while feeling more overwhelmed, than those using the basic UI.

**Interpretation:**

Our findings indicate that an optimized UI based on UX design principles, in comparison to a basic UI comprising elementary UI features, do not affect overall self-rated usability or treatment credibility but increases some measures of behavioral engagement with a digital intervention.

**Funding:**

Funded by the Government of Sweden, Ministry of Health and Social Affairs.

## Highlights

• The effect of user interface on engagement in internet interventions is unknown.
• We conducted a randomized trial of a problem-solving intervention.
• We investigated effects of two different user interfaces on treatment engagement.
• An optimized user interface increased behavioral engagement with a digital intervention.
• These results may guide development towards more effective interventions.

## 1 Introduction

### 1.1 Digital mental health services

Digital Mental Health Services (DMHS) are psychological services offered via the Internet, with the benefit of increasing access to mental health care (Titov et al., 2019). Currently, the most widespread form of DMHS is Internet-delivered cognitive behavioral therapy (ICBT) (Titov et al., 2019), conventionally provided in a clinician-guided digital self-help format (Andersson et al., 2008). ICBT may also be delivered without clinician-guidance, namely in a self-guided format (Titov et al., 2015; Dear et al., 2016; Schueller and Torous, 2020).

When providing DMHS, it is crucial that participants use and engage with the systems where the care is delivered, in order to access key interventions (Ossebaard et al., 2012; Chandrashekar, 2018) and benefit from treatment (Perski et al., 2017). In both clinician-guided and self-guided ICBT, insufficient engagement and adherence remains a problem (Doherty et al., 2012; Titov et al., 2013; Hedman et al., 2014; van Ballegooijen et al., 2014), leaving many patients not fully exposed to the treatment. Thus, increasing treatment engagement should be highly prioritized within DMHS.

It has been suggested that an optimized user interface (UI), that takes user experience (UX) into account, should be beneficial for the engagement with digital mental health interventions (Lemon et al., 2020). When aiming for enhanced UX, it has been suggested that it is vital to apply design principles that make the UI simple and intuitive to use, in order to facilitate user engagement (Bakker et al., 2016; Chandrashekar, 2018; Lemon et al., 2020). Recommendations regarding what such design should comprise include to limit text content to manageable chunks, to use internal triggers encouraging engagement, to use reminder functions, and to enable access to optional content through “learn more”-options (Bakker et al., 2016). Furthermore, using persuasive design principles, including task support such as a stepwise presentation of intervention related content, has been preliminarily linked to larger effects on depression in unguided digital interventions for depression (McCall et al., 2021). Additionally, implementing design principles that help users to carry out the primary task, such as reducing complex tasks to simpler ones or guiding the users through a process, have been suggested to be important for achieving durable behavior change (Oinas-Kukkonen and Harjumaa, 2009; Kelders et al., 2012).

Several studies have looked into or discussed whether an improved UI is crucial for treatment engagement within digital mental health interventions (Andersson et al., 2009; Kelders et al., 2012; Knowles et al., 2014; Ludden et al., 2015; Nitsch et al., 2016; Bauer et al., 2018; Baumel and Kane, 2018; Wei et al., 2020; Yogarajah et al., 2020), but none has been able to ascertain a causal effect of UI on treatment engagement due to uncontrolled study designs. As such, the importance of UI design for treatment engagement in DMHS remain largely unknown (Lemon et al., 2020; Yogarajah et al., 2020), and the need for randomized controlled trials focusing on design principles for UIs has been highlighted (Bakker et al., 2016; Yogarajah et al., 2020). Despite the lack of data on the effect of UI on treatment engagement, resources are spent worldwide on the development and design of digital treatment platforms and their UIs (Beukes et al., 2016; Dryman et al., 2017; Bauer et al., 2018; Ospina-Pinillos et al., 2018).

### 1.2 Treatment engagement

Treatment engagement within DMHS has been conceptualized and measured in different ways (Perski et al., 2017; Ng et al., 2019). Within the field of computer science and human-computer interaction, engagement has been conceptualized as the subjective experience of interacting with a system, while in the field of behavioral science it has instead been conceptualized as the usage of the DMHS (Perski et al., 2017). This has led to a suggested two-part definition of engagement with DMHS, involving both the subjective experience of being engaged, and to what extent the service is being used (Perski et al., 2017).

To gain a better understanding of users' subjective experiences of engagement with a digital intervention, usability and treatment credibility have frequently been measured (Ng et al., 2019). Usability, or the perceived ease of use of a system, has been defined as “the degree to which a person believes that using a particular system would be free of effort” (Davis, 1989). Problems related to usability have been proposed to be linked to non-usage and drop-out of treatment (Eysenbach, 2005; Torous et al., 2018), which adds value to it being assessed in evaluations of treatment engagement. Treatment credibility, which refers to a patient's belief in a treatment or intervention (Alfonsson et al., 2016), has also been linked to usage and engagement. High levels of treatment credibility have shown to be positively associated with both adherence and treatment outcome, as well as negatively associated with drop-out rates (El Alaoui et al., 2015; Alfonsson et al., 2016). Evaluating whether a digital intervention is presented in a trustworthy fashion and is perceived as likely to be helpful is thus also vital to understand more about treatment engagement.

When evaluating to what extent a DMHS is being used, behavioral engagement is conventionally measured through the completion of treatment modules and/or assignments, as well as through the number of logins on a DMHS (Alberts et al., 2018). Measures of behavioral engagement with the intervention can thus be seen as a useful complement to standardized questionnaires when investigating treatment engagement (Perski et al., 2017). Interventions focusing on problem solving (Cuijpers et al., 2018) could be suitable for examining behavioral engagement since problem solving is a universally useful skill, allowing participants to be recruited without strict exclusion criteria. Problem-solving interventions are well-examined, at least as a treatment for major depression, with similar effect sizes on symptoms of depression as other structured psychological interventions (Cuijpers et al., 2018).

### 1.3 Aim

The aim of this study was to investigate if treatment engagement differs between an optimized UI and a basic UI in a self-guided digital problem-solving intervention constructed for the Swedish general public experiencing practical and/or emotional problems during the coronavirus disease 2019 (COVID-19) pandemic. We planned to measure treatment engagement by 1) self-rated usability, 2) self-rated treatment credibility, and 3) behavioral engagement with the intervention. We hypothesized that the UIs would differ on these three outcomes, in favor of the optimized UI.

## 2 Material and methods

### 2.1 Setting and study design

The study was a randomized controlled trial investigating differences in treatment engagement between an optimized UI and a basic UI for DMHS. The study was approved by the Swedish national ethical review board (ID: 2020-02739) and retrospectively registered on ClinicalTrials.gov (ID: NCT04677270) 2020-12-21.

### 2.2 Participants and recruitment

To reach a broad population with a varying degree of mental health status and digital expertise, the recruitment was conducted among the Swedish general public during the COVID-19 pandemic. Sample size was determined based on power calculations for the primary outcome analyses, with 80% power and a standardized mean difference of 0.3. An additional 10% was planned to be recruited, to account for possible missing follow-up data.

Participants were recruited via ads on social media between August 26th and October 6th 2020. The recruitment was ongoing until the planned number of participants had enrolled in the study. Inclusion criteria were 1) 16 years old or above, and 2) self-reported practical and/or emotional problems experienced during the COVID-19 pandemic. No exclusion criteria were specified. Follow-up was conducted during the autumn of 2020, the week after the participants finished the intervention.

### 2.3 Procedures

Study registration and assessment was self-administered online on a secure platform. All participants provided written informed consent digitally to take part in the study. A total of 399 individuals from the general public registered to the study. Only two participants were excluded from the study, due to not meeting the second inclusion criterion (i.e., not experiencing any problems during the COVID-19 pandemic). Thus, a total of 397 participants were included and randomized (1:1) to use a self-guided digital problem-solving intervention, constructed for practical and emotional problems during the COVID-19 pandemic, either with an optimized UI (*N* = 198) or a basic UI (*N* = 199).

The randomization procedure was handled by an administrator blinded to the conditions and not otherwise involved in the study, using a custom R script. A blocking method was used, first generating the allocation numbers using a uniform distribution, ensuring both groups were balanced. The allocation numbers were thereafter divided into eight balanced blocks. For each individual, a number was drawn from the current block using a uniform distribution for all remaining numbers in the block. The allocation number was given to the study coordinator (AH), who was responsible for both enrollment of participants and assignment of participants to allocated UI. Furthermore, participants were blinded to which UI they were allocated to. Since no therapist or assessor was involved in the study, no such person was blinded.

**Fig. 1:**
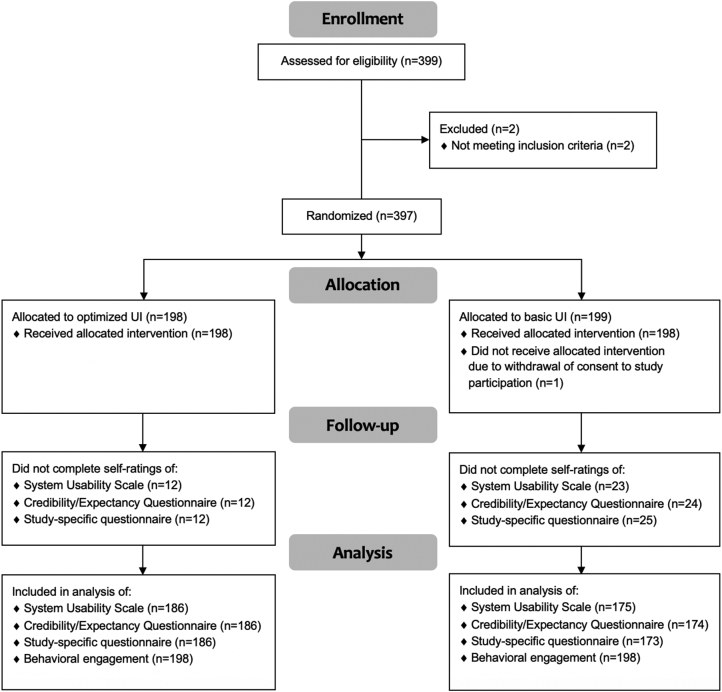
Flowchart.

After randomization, participants accessed the allocated UI and problem-solving intervention for a period of one week. One participant, randomized to use the basic UI, was excluded from the analyses due to withdrawal of consent for study participation after randomization. See Fig. 1 for the study flowchart.

### 2.4 Intervention

For both UIs, a problem-solving intervention was built in a secure digital treatment platform, and could be accessed via both computer and mobile devices connected to the Internet. The content was based on an already existing digital problem-solving intervention used as a component in ICBT in Swedish regular health care for individuals with major depression (Hedman et al., 2014), and was adapted for self-guidance in both UIs.

The intervention in both UIs comprised psychoeducational texts, treatment rationale, examples of problems and suggestions of solutions, illustrative pictures, instructions and problem-solving exercises. The intervention consisted of approximately 4800 words, both in the optimized and basic UI. In both UIs, all content was presented in Swedish. Reminders regarding to remember using the intervention were sent to all participants by external text messages during the week of access.

### 2.5 User interfaces

**Fig. 2:**
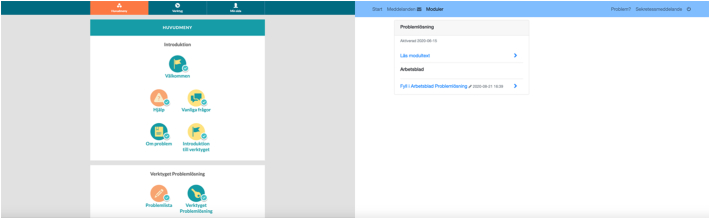
Main menu in optimized UI (left) versus basic UI (right).

**Fig. 3:**
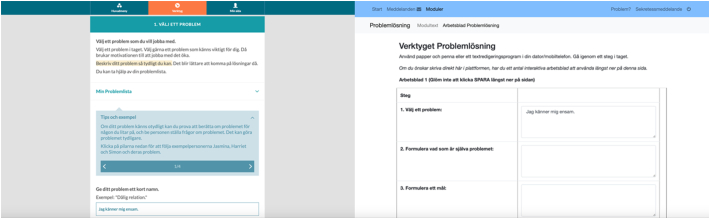
Part of the intervention in optimized UI (left) versus basic UI (right).

The optimized UI was developed in collaboration with paid expert consultants from the private sector specialized in UX of digital health care applications, in order to make the UI simple, intuitive and to some extent more automated. The basic UI was analogous with a UI used for over a decade in Swedish regular health care (Hedman et al., 2014) comprising elementary interface features and less automation. See Table 1 for a summary of differences between the UIs, and Fig. 2, Fig. 3 for examples on how the UIs differed visually.

**Table 1:** Differences between the UIs.

Design feature	Optimized UI	Basic UI
Type of responsive design	Mobile-first	Desktop-first
Navigation menu	Three main sections presented with headlines and pictograms	One main section presented with headline only
Division of content	Subsections, pages and expandable learn-more options	Subsections
Presentation of content	Presented in small chunks	All content in one scrollable page
Exercises	Stepwise fashion	Full intervention presented on one page
Instructions	Separate instructions at each step	All instructions provided on one page
Examples and suggestions	Presented within expandable containers	Presented directly as part of the rest of the content
Automated features	Text entered in previous steps in exercises automatically synched to upcoming steps when relevant; Automatic pop-ups within exercises containing control questions and encouraging words; New subsections based on initiated problem-solving attempts	None

UI, user interface.

#### 2.5.1 Type of responsive design

Since mobile device usage has increased among the general public (Parasuraman et al., 2017), the optimized UI was built with the responsive design method mobile-first, which means that the product design began from the mobile end before expanding to a desktop version. The basic UI was built with the responsive design method desktop-first, meaning that the design was primarily built for full size desktop screens and to be used with a physical keyboard and a mouse or trackpad.

#### 2.5.2 Navigation menu

Both UIs contained a navigation menu. In the optimized UI, the intervention related content was divided into three main sections within the navigation menu. The main sections were presented with headlines and accompanying pictograms aiming to visually differentiate the main sections. In the basic UI, all intervention related content was found under one main section, presented with a headline.

#### 2.5.3 Division of content

In both UIs, main sections were further divided into subsections containing the actual content. In the optimized UI, the first main section contained all information needed before working with the exercises, and was divided into seven subsections with headlines accompanied by pictograms. Within these subsections, content was further divided into pages in order to limit the amount of content displayed simultaneously. In some pages, expandable learn-more options were available. The second main section contained the problem-solving exercises, and new subsections were automatically created when problem-solving attempts were initiated. The third main section contained trial related information, and was divided into subsections presenting an overview of the trial period, a function for reporting system related problems, a re-presentation of the study privacy notice, and a log-out function.

In the basic UI, the main section was divided into two subsections. The first subsection contained all intervention related information, and the second subsection contained the problem-solving exercises. Content was not further divided. However, an overview of the trial period, a function for reporting system related problems, a re-presentation of the study privacy notice, and a log-out function was available separate from the main menu. A default message function was visible in the basic UI, but participants were informed of that the function was turned off during the trial.

#### 2.5.4 Presentation of content

In the optimized UI, the content was presented in small chunks, which was made possible by the several subsections, pages and learn-more options built into the design. In the basic UI, all content was presented at once in a scrollable page.

#### 2.5.5 Exercises

The problem-solving exercises were displayed in a stepwise fashion in the optimized UI, with each new step depending on information entered in previous steps. The intention with the stepwise fashion was to make the exercises simple and intuitive to use. In the basic UI, the exercises were presented in digital worksheets arranged one after another. The worksheets were designed to imitate exercises displayed on a paper sheet, and designed as tables with several rows and columns presented all together on one page.

#### 2.5.6 Instructions

For each new step in the optimized UI, separate instructions were provided. In the basic UI, all instructions were given at once, on one page, ahead of the exercises.

#### 2.5.7 Examples and suggestions

Through the content and the exercises within the optimized UI, expandable containers were provided, comprising examples and suggestions. In the basic UI, examples and suggestions were provided directly as part of the rest of the content.

#### 2.5.8 Automated features

The optimized UI comprised automated features. Firstly, within the problem-solving exercises, the text entered by the participant was automatically synched to the upcoming steps when appropriate. Secondly, automatic pop-ups were built into the exercises, exposing the participant to control questions prompting behaviors such as making sure previous exercises were completed. Some of the pop-ups also contained encouraging words, aiming to increase the motivation to use the intervention. Thirdly, new subsections were automatically generated when problem-solving attempts were initiated. The basic UI did not comprise any automated features.

### 2.6 Measurements

The registration procedure comprised a digital questionnaire concerning demographics and inclusion criteria, and two self-assessed short-scales, Patient Health Questionnaire-2 (PHQ-2) (Kroenke et al., 2001; Löwe et al., 2005) and Generalized Anxiety Disorder-2 (GAD-2) (Kroenke et al., 2007; Löwe et al., 2010), measuring symptoms of depression and anxiety respectively. These short-scales were not used either for inclusion or as an outcome measure, but were administered to assess possible clinical symptom burden among participants.

After the one week of access to the allocated UI and problem-solving intervention, participants completed the standardized and self-assessed questionnaires System Usability Scale (SUS) (Lewis, 2018), measuring the perceived usability of a system, and a five-item version of the Credibility/Expectancy Questionnaire (CEQ) (Borkovec and Nau, 1972), measuring treatment credibility. These were the primary outcome measures. Participants also completed a study-specific questionnaire concerning perceived UX, with a four-point scale (0–3) ranging from strongly disagree to strongly agree. This questionnaire consisted of four questions focusing on whether 1) the intervention was perceived as likable, 2) the intervention was easy to understand, 3) examples given felt relevant, and whether 4) functionality and information contributed to the participant feeling overwhelmed. Participants were invited to write a free-text comment to their responses on the questionnaire, and were also offered to describe whether anything in particular would make the intervention easier and more attractive to use. Measures of behavioral engagement were retrieved from the treatment platform and included 1) the number of logins to the platform, 2) the number of participants who used the intervention at least once, 3) the number of problem-solving attempts initiated, 4) the total number of generated solutions, 5) the mean number of generated solutions per initiated problem-solving attempt, and 6) the number of participants who completed at least one evaluation of a problem-solving attempt. In addition to this, the type of device used for logging into the platform (mobile device and desktop device respectively) was assessed.

### 2.7 Data analysis

Independent *t*-tests with 95% confidence intervals (CI) were conducted for all continuous variables. Chi-square tests for independence were conducted for all categorical variables, reported with odds ratios using 95% confidence intervals. Participants who did not complete the SUS, CEQ and/or study-specific questionnaire could not be included in the analyses of those data. Behavioral engagement data were available for all participants, and thus no participant was excluded from those analyses.

Illustrative free-text comments from the study-specific questionnaire were selected by the authors AH and MK, resulting in a compilation of quotes. These quotes were then translated from Swedish to English, also by authors AH and MK.

## 3 Results

### 3.1 Demographics

The majority of participants were female, highly educated and had symptoms of either depression, anxiety, or both. See Table 2 for complete sample characteristics.

**Table 2:** Baseline characteristics of participants who used the optimized UI and the basic UI respectively.

Variable	Optimized UI (N = 198)	Basic UI (N = 198)	Total (N = 396)
Female gender, n (%)	176 (89%)	176 (89%)	352 (89%)
Age, mean (SD) [range]	40 (12) [18–79]	40 (13) [17–74]	40 (13) [17–79]
In a relationship, n (%)	126 (64%)	120 (61%)	246 (62%)
Occupational status, n (%)			
Employed full time	105 (53%)	95 (48%)	200 (51%)
Employed part-time	19 (10%)	22 (11%)	41 (10%)
Student	37 (19%)	31 (16%)	68 (17%)
Parental leave	2 (1%)	5 (3%)	7 (2%)
Unemployed	16 (8%)	18 (9%)	34 (9%)
Long-term sick leave	7 (4%)	14 (7%)	21 (5%)
Retired	12 (6%)	13 (7%)	25 (6%)
Education, n (%)			
Primary school	3 (2%)	4 (2%)	7 (2%)
Secondary school	40 (20%)	38 (19%)	78 (20%)
University	155 (78%)	156 (79%)	311 (79%)
Possible major depression PHQ-2 ≥ 3, n (%)	123 (62%)	112 (57%)	235 (59%)
Possible generalized anxiety GAD-2 ≥ 3, n (%)	125 (63%)	111 (56%)	236 (60%)
Concurrent possible depression and anxiety, n (%)	100 (51%)	80 (40%)	180 (45%)
Either possible depression, anxiety, or both, n (%)	148 (75%)	143 (72%)	291 (73%)

UI, user interface; SD, standard deviation; PHQ-2, Patient Health Questionnaire-2; GAD-2, Generalized Anxiety Disorder-2. There were no significant differences between participants who used the different UIs on any baseline characteristic when testing with t-tests for continuous data and chi-square tests for categorical data.

### 3.2 Type of device used

Of a total of 1793 logins into the platform to both UIs, 1259 (70.2%) logins were from a mobile device, while 534 (29.8%) logins were from a desktop device. The proportion of logins by mobile device or desktop device did not differ by UI (mobile device in optimized UI *n* = 656/930 (70.5%), mobile device in basic UI *n* = 603/863 (69.9%)), χ2 (1, *N* = 1793) =0.07, *p* = .798, odds ratio = 1.03, 95% CI [0.84, 1.26].

### 3.3 Missing data

Among those who used the optimized UI, 12 participants (6%) had missing values on SUS, CEQ and the study-specific questionnaire. The corresponding numbers for those who used the basic UI were 23 participants (11.6%) on SUS, 24 participants (12.1%) on CEQ, and 25 participants (12.6%) on the study-specific questionnaire. There was a significant difference between groups in missing data on the study-specific questionnaire, with a greater risk of missing data among those who used the basic UI (*n* = 25/198, 12.6%) in comparison to those who used the optimized UI (*n* = 12/198, 6.1%), χ2 (1, *N* = 396) =4.29, *p* = .038, odds ratio = 2.24, 95% CI [1.09, 4.6].

### 3.4 Usability, treatment credibility and study-specific questionnaire

There were no significant differences between the UIs in ratings of usability, as measured by SUS (t(353.32) = −1.18, *p* = .240), or treatment credibility, as measured by CEQ (t(354.41) = −0.49, *p* = .625). On the study-specific questionnaire, no significant differences were shown between the UIs on the items concerning whether the intervention was perceived as likable (t(348.39) = 0.47, *p* = .639), or whether examples given felt relevant (t(348.21) = −1.12, *p* = .264). However, the UIs significantly differed on the item concerning whether the intervention was easy to understand (t(351.58) = −2.32, *p* = .021), in favor of the optimized UI, and on the item concerning whether functionality and information contributed to the participant feeling overwhelmed (t(336.88) = −2.37, *p* = .018), with participants who used the optimized UI again scoring higher, i.e., feeling more overwhelmed. Table 3 shows the results for the respective UIs on SUS, CEQ and the study-specific questionnaire.

**Table 3:** Outcomes on SUS, CEQ and study-specific questionnaire for the optimized UI and the basic UI respectively.

Variable	Optimized UI		Basic UI		Mean difference (95% CI)	*p*-Value
	N	Mean (SD)	N	Mean (SD)		
SUS	186	65.32 (18.74)	175	62.91 (20.02)	2.41 (−1.61, 6.43)	0.240
CEQ	186	26.43 (11.79)	174	25.81 (12.19)	0.62 (−1.87, 3.11)	0.625
Study-specific questionnaire	186		173			
Likable		1.34 (0.83)		1.39 (0.91)	−0.04 (−0.22,	0.639
Easy to understand		2.08 (0.78)		1.88 (0.82)	0.14)	0.021
Relevant examples		1.79 (0.78)		1.69 (0.85)	0.20 (0.03, 0.36) 0.10 (−0.07, 0.27)	0.264
Overwhelmed		1.88 (0.87)		1.64 (1.03)	0.24 (0.04, 0.44)	0.018

UI, user interface; CI, confidence interval; SD, standard deviation; SUS, System Usability Scale; CEQ, Credibility/Expectancy Questionnaire.

In the free-text comment sections in the study-specific questionnaire, participants reflected on positive and negative aspects of the UI they had used, as well as on suggestions of possible improvements of the UI. See Table 4 for selected illustrative quotes.

**Table 4:** Illustrative quotes regarding the UIs from participants who used the optimized UI and the basic UI respectively.

Item		Optimized UI	Basic UI
Likable	Positive aspect	“It was great to work with all steps within the intervention, and to complete one step at a time before heading to the next one. It was also good that I was able to return to previous steps and that the information I had entered was editable.” ID 101	“It is easy to use, and it is designed in a way that helps you to formulate problems as well as think of different solutions.” ID 112
	Negative aspect	“I think that there were too many steps. To set goals felt unnecessary. It complicated the use.” ID 102	“It was hard to use the intervention digitally, due to small text boxes and bad overview.” ID 113
Easy to understand	Positive aspect	“The instructions were well formulated, and it was evident what was expected of me.” ID 103	“I think that the instructions were very clear and simple.” ID 114
	Negative aspect	“I would have preferred a simpler and more compact version of the intervention. It felt unnecessarily complex and my impression was that it took too long before I actually got somewhere.” ID 104	“The intervention is a bit bulky and old-fashioned. A more user-friendly system would have been good!” ID 115
Relevant examples	Positive aspect	“Great examples. Many different types of problems are covered and you get a good understanding of how to think. It was good that the examples could be unfolded when not needed, so that they did not obstruct working with the intervention.” ID 101	“I returned to the examples several times. The examples describing simple problems, such as cleaning, were good since they made me not feel stupid about my own problems, and they made it easy to understand what to do.” ID 116
	Negative aspect	“I understand the purpose of including examples, but the examples are not that advanced or difficult.” ID 105	“I think that the examples should be optional and possible to expand. It is way too much text as it is now.” ID 117
Overwhelmed	Positive aspect	“As with all new digital tools, it takes a while to get used to it, as with everything you do for the first time. But it is not difficult at all. It is very logically described. One step at a time. Great!” ID 101	“Extensive introduction. But when one had managed to finished it, it was clear to me what I was going to do.” ID 118
	Negative aspect	“The introduction was too long and complicated.” ID 106	“There was too much information in the beginning, and everything was presented at once. It felt overwhelming.” ID 119
Suggestions of possible improvements		“Daily notifications reminding the user that it is time to work with the intervention.” ID 107 “The possibility to print the content.” ID 108 “Supervision and follow-up with a real person. Feedback and support.” ID 109 “Lists with suggestions of problems to choose from, together with suggestions of solutions to those problems.” ID 110 “Video clips comprising instructions.” ID 111	“Less amount of text. Information divided it into foldable sections.” ID 117 “Better adaptation to mobile phones, to decrease the amount of scrolling needed.” ID 120 “More suggestions on how to work with problems.” ID 121 “Help-texts, and pop-ups with explanations.” ID 122 “Information presented in a more interactive manner.” ID 123

Same row does not indicate that the quote is from the same participant.

### 3.5 Behavioral engagement

On the behavioral engagement measures, there was a significant difference between the UIs in number of participants who used the intervention at least once, with more participants using the intervention in the group who used the optimized UI (*n* = 142/198, 71.7%) in comparison to the group who used the basic UI (*n* = 99/198, 50%), χ2 (1, *N* = 241) = 18.7, *p* < .001, odds ratio = 2.54, 95% CI [1.67, 3.85]. Furthermore, the UIs significantly differed, in favor of the optimized UI, on total number of generated solutions (t(385.20) = −3.99, *p* < .001) as well as mean number of generated solutions per initiated problem-solving attempt (t(295.54) = −7.25, *p* < .001). The UIs did however not significantly differ on number of logins to the platform (t(383.13) = −1.82, *p* = .070) or number of problem-solving attempts initiated (t(294.20) = 1.12, *p* = .265). Moreover, there was no significant difference between the UIs in number of participants who completed at least one evaluation of a problem-solving attempt (optimized UI *n* = 59/198 (29.8%), basic UI *n* = 44/198 (22.2%)), χ2 (1, *N* = 396) = 2.57, *p* = .109, odds ratio = 1.49, 95% CI [0.94, 2.34]. Table 5 shows the results for the respective UIs on the behavioral engagement measures.

**Table 5:** Outcomes on behavioral engagement measures for the optimized UI and the basic UI respectively.

Variable	Optimized UI (N = 198), mean (SD)	Basic UI (*N* = 198), mean (SD)	Mean difference (95% CI)	p-Value
Number of logins to the platform	4.70 (2.0)	4.36 (1.69)	0.34 (−0.03, 0.7)	0.070
Number of problem-solving attempts initiated	1.05 (0.93)	1.21 (1.81)	−0.16 (−0.45, 0.12)	0.265
Total number of generated solutions	3.37 (3.79)	1.95 (3.25)	1.41 (0.72, 2.11)	<0.001
Mean number of generated solutions per initiated problem-solving attempt	2.38 (2.51)	0.92 (1.30)	1.45 (1.06, 1.85)	<0.001

UI, user interface; SD, standard deviation; CI, confidence interval.

### 3.6 Sensitivity analysis

Please see the supplement for an additional sensitivity analysis conducted only on participants who used the intervention at least once. In the sensitivity analysis, the UIs did not significantly differ on ratings of SUS or CEQ. On the study-specific questionnaire, the UIs differed only on the item concerning whether the intervention was easy to understand, in favor of the optimized UI. On the behavioral measures, there was a significant difference between the UIs on number of problem-solving attempts initiated, in favor of the basic UI, and on mean number of generated solutions per initiated problem-solving attempt, in favor of the optimized UI.

## 4 Discussion

### 4.1 Main discussion

The aim of this study was to investigate if treatment engagement differs depending on type of UI in a self-guided digital problem-solving intervention. We tested this in a randomized controlled trial where participants got to use either an optimized UI, based on UX design principles together with some automated features in order to make the UI simple and intuitive, or a basic UI, comprising elementary interface features. There were no differences between the UIs in ratings of usability or treatment credibility, contrary to our hypothesis. However, in line with our hypothesis, the results indicate that participants who used the optimized UI were more behaviorally engaged with the intervention in comparison to those who used the basic UI. Since low adherence and treatment engagement is a critical matter when it comes to DMHS (Hedman et al., 2015; Hadjistavropoulos et al., 2016), these findings may act as a guidance for healthcare providers developing and improving digital mental health services, and possibly contribute to enhanced treatments.

The fact that the UIs did not differ in ratings of usability and treatment credibility, but in behavioral engagement, in favor of the optimized UI, underscores the importance of complementing self-rated treatment engagement with behavioral engagement data (Perski et al., 2017). One possible explanation to why the ratings of usability and treatment credibility did not differ between the UIs may be that both UIs had sufficient technical and visual features, resulting in both UIs being perceived as adequate on these measures. A digital healthcare intervention, presented in a similar fashion as in the basic UI, was in previous study rated as sufficient concerning user satisfaction and usability aspects (Beukes et al., 2016). In other words, a UI might not need to be optimized in order for users to perceive it as sufficient. However, since the optimized UI nonetheless led to increased behavioral engagement, the stepwise design fashion of the exercises along with the other UX optimized features seem to have been important for impacting the users' behaviors. This is in line with the suggestion that taking UX and simple and intuitive design into consideration is vital for behavioral engagement (Bakker et al., 2016).

The results regarding participants feeling more overwhelmed by using the optimized UI might be due to that the optimized UI did actually require more of the user. Participants who used the optimized UI were however not only more behaviorally engaged than the other group, but did also rate the intervention as easier to understand. This could be interpreted as even though the optimized UI was perceived as more overwhelming, it did apparently not reach a level of overwhelmingness preventing participants from understanding or engaging with the exercises. An illustrative quote in line with this from a participant who used the optimized UI is the following: "As with all new digital tools, it takes a while to get used to it, as with everything you do for the first time. But it is not difficult at all. […]” This again underscores the importance of putting self-reported data regarding engagement and satisfaction in relation to behavioral engagement data (Perski et al., 2017). While logins on the platform did not differ between the UIs, the usage of the intervention as well as the number of generated solutions did. With regards to these results, it should be highlighted that it has been argued that behavioral engagement with the *treatment* should be of higher interest than engagement with the *technology* in the context of DMHS (Coyle and Doherty, 2009). That is, the number of logins or other engagement with the technical platform is not as important as the participants' completion of activities related to their real-world challenges.

There was no difference between the UIs in number of problem-solving attempts initiated. Since the participants who used the optimized UI generated more solutions than the participants who used the basic UI, these results could weighed together be interpreted as the optimized UI leading to further engagement with the intervention. It is nevertheless unclear if this resulted in more problems solved or better solutions. Even if there was no difference between the UIs in number of participants who completed at least one evaluation of a problem-solving attempt, we do not know the success rate of the participants' problem-solving attempts. However, since one aim of the problem-solving intervention is to practice problem-solving skills such as reflecting on different solutions (Cuijpers et al., 2018), the results still favors the optimized UI.

In the sensitivity analysis on participants who used the intervention at least once, participants who used the basic UI initiated a significantly larger number of problem-solving attempts. This is probably due to selection bias in the sensitivity analysis, i.e. when participants who never used the intervention were excluded from the analysis, the result falsely gives the impression that individuals using the basic UI were more active. The result could however perhaps be affected by the basic UI having a possible lower threshold for initiating new problem-solving attempts before the completion of already started attempts.

In the compilation of quotes from the free-text section in the study-specific questionnaire, several suggestions of possible improvements are highlighted. Apart from the suggestions concerning technical or content related improvements, supervision and support from a real person was requested several times and is highlighted in one quote. Based on our reading of all participants' free-text comments, we believe that a self-guided digital format should not aim to replace clinician-guided digital treatment or face-to-face treatment, but instead complement these formats, offering a format for those who prefer structured psychological self-care. These readings of the free-text comments are however preliminary, and future analyses of the comments with a complete categorization of participants' viewpoints on guidance are needed.

In summary, it is likely that an optimized UI based on UX design principles can be helpful when aiming for increased behavioral engagement. Since it has been indicated in a recent meta-analysis that self-guidance in DMHS is associated with a lower adherence rate than clinician-guidance (Karyotaki et al., 2021), an improved UI might be even more important for self-guided DMHS, in order to reduce possible practical or content related obstacles that in a clinician-guided format could be facilitated by the clinician.

### 4.2 Limitations

This study had several limitations that need to be acknowledged. Firstly, due to lack of resources, the problem-solving intervention was not given as a full-length intervention in this study, which could complicate generalization of the results to treatment settings where participants use an intervention during a longer period of time. Secondly, the sample in this study was chosen to represent a broad population with varying mental health status. This impacts the possibility to generalize the results to a psychiatric or primary care setting, even though a majority of the sample did experience clinically relevant symptoms of depression and/or anxiety. Thirdly, regarding behavioral engagement, it is not certain whether the measured level of engagement within the platform corresponds to the level of engagement in real life. Some participants might have engaged more than digitally documented. However, since behavioral engagement within DMHS is conventionally measured through behaviors within the DMHS, the results are highly relevant to this field. Fourthly, there are some consequences of missing data. On the study-specific questionnaire, significantly fewer participants in the group using the basic UI completed the questionnaire. This may have biased the analyses of this measure. For example, the mean rating of whether the intervention was easy to understand might have been affected by participants in the basic UI who possibly found the intervention hard to understand but did not respond to this measure. Furthermore, there were no significant differences in missing data between groups on the SUS or CEQ, although there was a significant difference concerning usage of the intervention at least once, in favor of the optimized UI. This indicates that a larger proportion of participants in the group who used the basic UI, in comparison to the group who used the optimized UI, completed the evaluations of usability and treatment credibility without having used the intervention. It is however possible that some non-using participants actually examined the content of the intervention before evaluating it, even though they did not use the intervention. This would counteract the potential risk of bias introduced by participants rating usability and treatment credibility without having used the intervention. Lastly, although 397 participants constitute a relatively large sample size given the study time frame, more participants would have helped making these results more robust.

### 4.3 Clinical implications

The results from this study indicate that an optimized UI based on UX design principles contributes to increased behavioral engagement. Since insufficient treatment engagement is an acknowledged problem within DMHS, the hands-on guidance for UI design provided in this paper, in the form of design features used in the optimized UI, could be used by healthcare providers looking for guidelines when developing or improving UIs for DMHS. Furthermore, since most logins were from mobile devices, a practical implication is that mobile-first design methods should be used to facilitate mobile device usage of the intervention.

### 4.4 Future research

Future randomized controlled trials replicating this study protocol, with a similar intervention as well as with complete treatment protocols, are needed. Another future possibility is to explore the effect of UIs on treatment engagement in self-guided versus clinician-guided treatment formats, and on a clinical population with symptoms of depression and anxiety as outcome measures. Based on our experiences from this study, we would recommend future studies to use behavioral engagement as primary outcome measure, complemented by self-rated data, and not the other way around. Moreover, there are complementary assessment tools available to evaluate user engagement indicators, which could be used in future studies. For a list, see the paper by Ng et al. (2019).

## 5 Conclusions

This study covers, to our knowledge, for the first time in research on digital mental health interventions a randomized controlled evaluation of the effect of an optimized versus basic UI on treatment engagement. Our findings indicate that self-rated usability and treatment credibility may not be affected by whether the UI is optimized or not. However, an optimized UI may nevertheless contribute to an increased level of some measures of behavioral engagement with the intervention. More specifically, participants who were allocated to the optimized rather than the basic UI did not log in more frequently, but when they did, they engaged significantly more with the intervention by generating more solutions to their problems.

Since treatment engagement is a cornerstone when it comes to improvement during treatment, the results from this study could help guide decisions concerning development and improvement of UIs for DMHS to possibly enhance treatment outcome.

## Role of the funding source

The funding source of this study did not have any role in the study design; the collection, analysis, or interpretation of data; the recruitment; the writing of the paper; or in the decision to submit the paper for publication.

## Data sharing

Deidentified participant data will be made available on reasonable request to the corresponding author.

## CRediT authorship contribution statement

AH performed the literature search, the statistical analyses, and drafted the manuscript. AH and MK selected illustrative quotes. AH, MK and BL contributed to the acquisition of data. All authors, AH, EF, BL, VK, NL and MK, were involved in the study design, contributed to the interpretation of data, and have read, revised and approved the manuscript. AH, MK and BL verify the accuracy of the underlying data.

## Declaration of competing interest

BL is Shareholder of DahliaQomit AB, a company specializing in online psychiatric symptom assessment, and Hedman-Lagerlöf och Ljótsson psykologi AB, a company that licenses cognitive behavior therapy manuals. AH, EF, VK, NL and MK have no competing interests.

## Appendix A Supplementary data

Sensitivity analysis Image 1
