# Supplementary material for: The effect of user interface on treatment engagement in a self-guided digital problem-solving intervention: A randomized controlled trial
Source: Internet Interv. 2021 Aug 20;26:100448. doi: 10.1016/j.invent.2021.100448 (PMC8387893; doi:10.1016/j.invent.2021.100448)
Supplement: Supplementary file 1 — Sensitivity analysis [file mmc1.docx]

Supplement

Outcomes on SUS, CEQ and study-specific questionnaire for participants who used the intervention at least once with the optimized UI and the basic UI respectively

| **Variable** | **Optimized UI** | | **Basic UI** | | **Mean difference (95% CI)** | **p-value** |
| --- | --- | --- | --- | --- | --- | --- |
|  | **N** | **Mean (SD)** | **N** | **Mean (SD)** |  |  |
| SUS | 139 | 67.09 (18.73) | 95 | 65.82 (19.91) | 1.27 (-3.83, 6.37) | .624 |
| CEQ | 139 | 27.24 (11.89) | 95 | 26.39 (12.85) | 0.85 (-2.43, 4.12) | .610 |
| Study-specific questionnaire     Likable  Easy to understand  Relevant examples  Overwhelmed | 139 | 1.38 (0.85)  2.17 (0.81)  1.85 (0.80)  1.98 (0.84) | 95 | 1.43 (0.94)  1.96 (0.81)  1.67 (0.93)  1.78 (1.04) | -0.05 (-0.29, 0.19)  0.21 (0, 0.43)  0.18 (-0.06, 0.41)  0.20 (-0.05, 0.45) | .677  .048  .135  .122 |

UI, user interface; CI, confidence interval; SD, standard deviation; SUS, System Usability Scale; CEQ, Credibility/Expectancy Questionnaire.

Outcomes on behavioral engagement measures for participants who used the intervention at least once with the optimized UI and the basic UI respectively

| **Variable** | **Optimized UI (N=142), mean (SD)** | **Basic UI (N=99), mean (SD)** | **Mean difference (95% CI)** | **p-value** |
| --- | --- | --- | --- | --- |
| Number of logins to the platform | 5.15 (2.07) | 4.94 (1.55) | 0.22 (-0.24, 0.68) | .357 |
| Number of problem-solving attempts initiated | 1.46 (0.78) | 2.41 (1.91) | -0.96 (-1.36, -0.55) | <.001 |
| Total number of generated solutions | 4.70 (3.71) | 3.91 (3.68) | 0.79 (-0.16, 1.74) | .104 |
| Mean number of generated solutions per initiated problem-solving attempt | 3.31 (2.38) | 1.84 (1.29) | 1.47 (1, 1.94) | <.001 |

UI, user interface; SD, standard deviation; CI, confidence interval.

Among those who used the intervention at least once, there was no significant difference between the UIs in the number of participants who completed at least one evaluation of a problem-solving attempt (optimized UI n=59/142 (41.5 %), basic UI n=44/99 (44.4 %)), χ2 (1, N=241) = 0.099, p=.753, odds ratio=0.89, 95 % CI [0.53, 1.49].
